# Geographical distribution of risk factors for invasive non-typhoidal Salmonella at the subnational boundary level in sub-Saharan Africa

**DOI:** 10.1186/s12879-021-06198-1

**Published:** 2021-06-05

**Authors:** Jung-Seok Lee, Vittal Mogasale, Florian Marks, Jerome Kim

**Affiliations:** International Vaccine Institute, SNU Research Park, 1 Gwanak-ro, Gwanak-gu, Seoul, 08226 South Korea

**Keywords:** Invasive non-typhoidal *Salmonella*, Risk factors for iNTS, Geographical variation of risk factors, Composite index for iNTS risk factors

## Abstract

**Background:**

Invasive non-typhoidal *Salmonella* (iNTS) is a growing health-concern in many parts of sub-Saharan Africa. iNTS is associated with fatal diseases such as HIV and malaria. Despite high case fatality rates, the disease has not been given much attention. The limited number of population-based surveillance studies hampers accurate estimation of global disease burden. Given the lack of available evidence on the disease, it is critical to identify high risk areas for future surveillance and to improve our understanding of iNTS endemicity.

**Methods:**

Considering that population-based surveillance data were sparse, a composite index called the iNTS risk factor (iNRF) index was constructed based on risk factors that commonly exist across countries. Four risk factors associated with the prevalence of iNTS were considered: malaria, HIV, malnutrition, and safe water. The iNRF index was first generated based on the four risk factors which were collected within a 50 km radius of existing surveillance sites. Pearson product-moment correlation was used to test statistical associations between the iNRF index and the prevalence of iNTS observed in the surveillance sites. The index was then further estimated at the subnational boundary level across selected countries and used to identify high risk areas for iNTS.

**Results:**

While the iNRF index in some countries was generally low (i.e. Rwanda) or high (i.e. Cote d’Ivoire), the risk-level of iNTS was variable not only by country but also within a country. At the provincial-level, the highest risk area was identified in Maniema, the Democratic Republic of Congo, whereas Dakar in Senegal was at the lowest risk.

**Conclusions:**

The iNRF index can be a useful tool to understand the geographically varying risk-level of iNTS. Given that conducting a population-based surveillance study requires extensive human and financial resources, identifying high risk areas for iNTS prior to a study implementation can facilitate an appropriate site-selection process in the future.

**Supplementary Information:**

The online version contains supplementary material available at 10.1186/s12879-021-06198-1.

## Background

*Samonella enterica*, a single bacterial species, is a major public health concern in many parts of the world. In terms of human disease-causing serovars, *Samonellae* are divided into typhoidal and non-typhoidal groups: (1) *Salmonella enterica* serovars Typhi, Paratyphi A, B, and C are often collectively considered as typhoidal *Salmonella*, (2) other serovars including *Salmonella enterica* serovar Typhimurium and *Salmonella enterica* serovar Enteritidis are grouped as nontyphoidal *Salmonella* (NTS) [1, 2]. Typhoidal *Salmonella* strains are human-restricted organisms that cause enteric fever. While enteric fever is generally found in immunocompetent children and adults who are exposed to environmental or behavioral risk factors, invasive non-typhoidal *Salmonella* (iNTS) disease is more associated with differential host susceptibility to invasive disease rather than environmental factors [2, 3].

Globally, population-based surveillance data for iNTS including disease burden estimates, are scarce compared to that for typhoid and paratyphoid. In 2010, Ao et al. estimated the global incidence and deaths of iNTS based on a systematic literature review and extrapolation using key risk factors which are associated with the disease [4]. The highest incidence rate was observed in Africa followed by Europe where a high number of cases were mostly driven by Russia, Ukraine, and Estonia. The World Health Organization (WHO) and Kirk et al. also updated the global burden of iNTS by calculating disability-adjusted life years (DALYs) [5, 6]. Based on these studies, the geographical distribution of the global iNTS burden was roughly demonstrated [7]. Similar to Ao et al., the global burden of iNTS expressed by DALYs was greatest in Africa. More recently, the Institute for Health Metrics and Evaluation (IHME) included the global burden of iNTS in its cause list for the first time and reported the highest incidence in sub-Saharan Africa as well [8]. The IHME study also indicated that compared to the two previous studies for the same year, their estimates were slightly higher than those by WHO [5] and significantly lower than those by Ao et al. [4]. While all previous efforts were helpful to understand the geographical variation of the disease, it should be noted that the source data underlying the models were sparse, which resulted in the extrapolation of limited surveillance data to neighboring countries or regions [4, 6–8]. This clearly indicates that more population-based surveillance data are urgently needed to improve our understanding of iNTS endemicity.

The current study attempts to identify potential high risk areas for iNTS at the subnational-level and to facilitate the site selection process for surveillance and vaccine studies. Because iNTS disease burden data are limited, the current study proposes the use of risk factors that are strongly associated with iNTS to identify high risk areas. Differential susceptibility of the host plays a major role in risk for iNTS disease [2]. As evidenced by previous studies [1, 3, 4, 9], the prevalence of iNTS is closely associated with the prevalence of malaria, HIV, and malnutrition. In addition, while it is debatable [10], several studies indicated that contaminated water sources could be partly associated with NTS as an environmental risk factor [11, 12]. Risk factors related to a disease are often available in a more standardized way at the country-level or subnational-level [13]. Incidence rates from a specific surveillance site in a country are frequently assumed to be representative of the whole country when estimating the burden of a disease or cost-effectiveness analyses. This approach is unavoidable due to the lack of population-based surveillance studies which require extensive human and financial resources. The use of standardized risk factors which are available at the subnational-level enables proper comparison of risk levels across countries or subnational boundaries in a more consistent manner. Understanding the endemicity of iNTS based on risk factors covers broader geographical areas where surveillance data are not yet available, and help researchers design future studies to examine incidence rates.

## Methods

### Data preparation

**Fig. 1:**
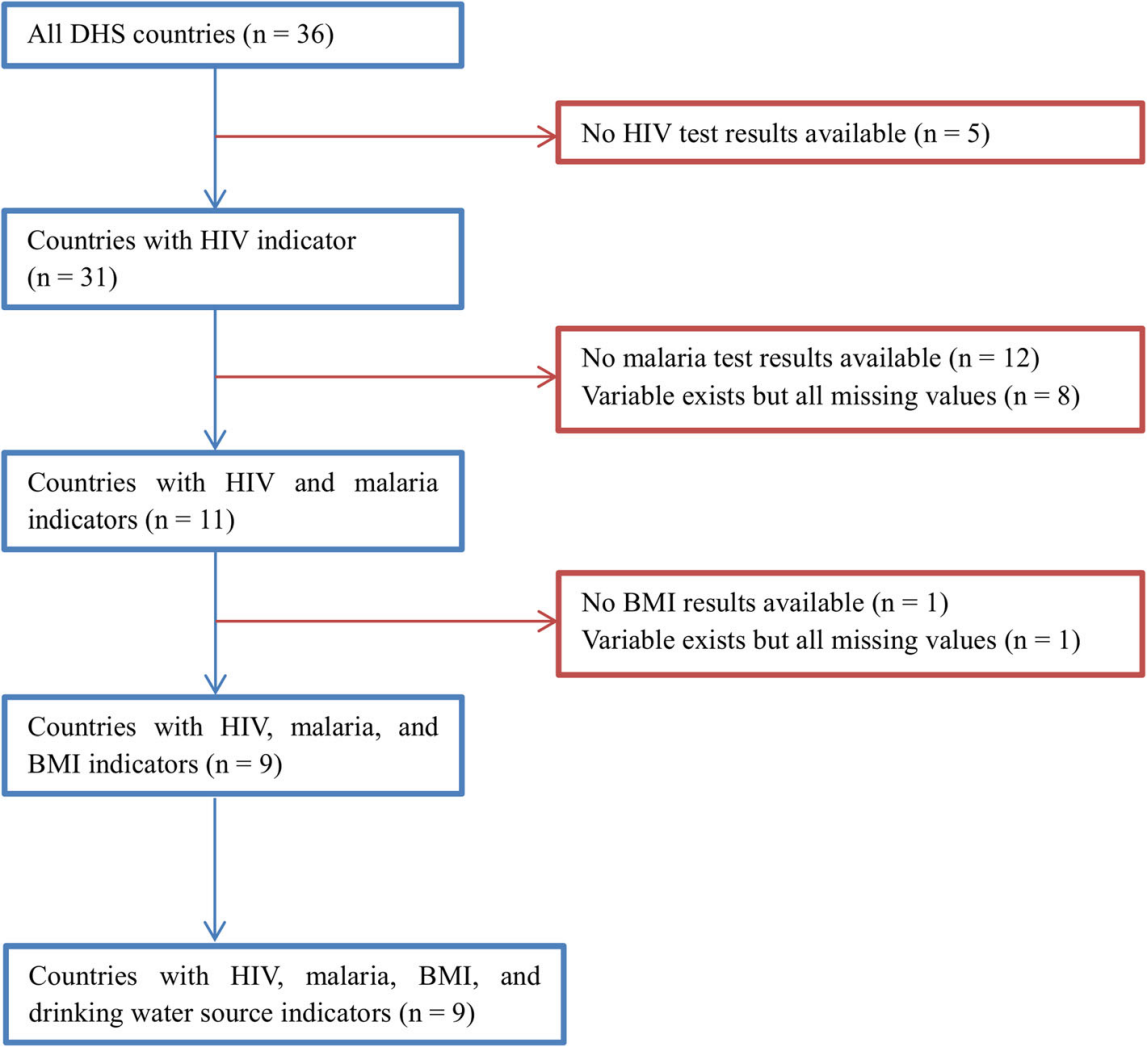
DHS datasets

The Demographic and Health Surveys (DHS) program has collected a wide range of population-health indicators in various countries [14]. Four indicators from the DHS program were considered: Malaria, HIV, Body Mass Index (BMI), and the main source of drinking water. All DHS datasets were first screened to ensure that the four indicators were included. Figure 1 shows the number of countries where all four risk factors were identified in the individual-level survey datasets.

The malaria rapid test results (HML35) and the blood test results (HIV03) were used to estimate the proportion of positive malaria and HIV episodes, respectively. The proportion of infected individuals was calculated by dividing the number of positive cases by the total number of people who were tested. In order to assess the level of malnutrition, the BMI indicator (HC73) was considered. The variable shows the standard deviations (SD) of BMI for those under the age of five. A Z-score cutoff value of − 2 SD was used to define malnutrition following the WHO criteria [15], and the proportion of undernourished children who were below the cutoff value was estimated. As an environmental factor, the main source of drinking water for household members (HV201) was adopted. Among the variable sub-options of HV201, drinking water sources which met the WHO criteria for improved drinking water source were selected [16]. The proportion of household members who have access to improved drinking water sources was estimated.

### iNTS risk factor (iNRF) index

Based on the four indicators, a composite index called iNTS Risk Factor (iNRF) index was created. Risk factor indices were previously developed for other diseases and proved useful to monitor risk levels and overall progress [13, 17, 18]. While the first three risk factors related to host susceptibility (malaria, HIV, and malnutrition) are associated with an increase in the prevalence of iNTS, access to improved drinking water source is linked to the opposite direction. Thus, the indicator for improved drinking water source was reversed prior to generating the index. The iNRF index was first constructed for validation where risk factors were collected for existing surveillance sites. Once validated, risk mapping was carried out by developing the index for all subnational boundary units of DHS countries where a complete set of the four risk factors existed. Each risk factor was first standardized by subtracting the mean and dividing by the standard deviation of each of two whole samples: (1) validation, and (2) risk mapping. The values were then averaged across the four risk factors and converted into a range from 0 (low risk) to 1 (high risk) [19]. The final iNRF index was generated by multiplying 100 for an easier interpretation [13, 17]. Given this index utilized the equal weighting scheme, an additional composite index was generated by assigning relative weights which were derived from a correlation of each risk factor with the proportion of iNTS (a convex combination of weight coefficients that sum to one).

### Validation

The iNRF index was first generated for selected locations where existing population-based surveillance studies were conducted. Among a series of previous literature on iNTS reviewed by Balasubramanian et al. [7], three population-based surveillance studies were selected [20–22]. These studies reported evidence on iNTS in multiple locations: Democratic Republic of Congo (7 provinces), Burkina Faso (Ouagadougou), Ghana (Asante), Senegal (Dakar), and Mali (Bamako). All three studies were chosen because they were conducted using primary data sources (neither extrapolation nor the use of secondary data), and there were corresponding DHS datasets where the four risk factors were commonly collected. In addition, two of the studies presented the number of iNTS episodes in a consistent manner by taking advantage of having multiple sites. Nonetheless, the studies by Lunguya et al. and by Tapia et al. were not designed to estimate the population-based incidence rates of iNTS [20, 22], and the other study by Marks et al. used adjusted-population denominators for the incidence rate calculation due to logistical reasons [21]. Thus, in order to make consistent comparisons between the existing studies and the iNRF index, the proportion of iNTS was estimated by dividing the number of the reported iNTS episodes by the total number of blood cultures obtained per site for the purpose of the current study.

Surveillance studies in the low- and middle-income country setting tends to be site-specific rather than to cover broader areas in a country. Therefore, the use of risk factors available at the country-level may not accurately explain the association with site-specific surveillance data. Given that the selected studies were carried out in specific locations within provinces in each country, it sounds more sensible to consider risk factors which are distributed in adjacent locations to the surveillance sites. Thus, prior to generating the iNRF index, the provincial boundary of each surveillance site and the geo-coordinates of the existing study sites were obtained. Given that DHS clusters contained geo-coordinates, all DHS clusters within a 50 km radius from each study site were selected to reflect more precise localization of the risk factors harbored where the surveillance studies were carried out. It should be noted that the dispersion of DHS survey clusters varied depending on the size of a country and other logistical constraints. Because no DHS clusters were observed within a 50 km radius from the geo-coordinates assigned to Kasai-Oriental province in the Democratic Republic of Congo, an 80 km radius was applied to this province. Both the iNRF index with an equal weight and the index with relative weights were then estimated for all study sites and compared with the proportion of iNTS per site. Pearson product-moment correlation was used to select the final index for further analyses, and the Bonferroni-adjusted significance of the correlation was tested. Sensitivity analyses were conducted to examine how varying radii (80 km and 100 km) would affect the correlations between the iNRF index and the proportion of iNTS observed from the surveillance sites.

### Risk mapping

The iNRF index was further estimated at the subnational boundary level for the final DHS countries where all four risk factor indicators were available (see Fig. 1). The DHS program provides sample weights which enable individual- or household-level datasets to be representative of the subnational boundary level (i.e. province or state) in a country. Thus, all individual-level datasets were weighted prior to estimating the iNRF index at the subnational boundary level. The index values were categorized into 10 risk-levels based on every 10th percentile for comparisons among provinces [17]. The final iNRF index values were mapped to identify potential high-risk areas for iNTS.

### Ethics approval and consent to participate

The current study does not contain any individual-level data which were directly collected for the purpose of the study, thus ethics approval and consent to participate were not required.

### Permission to access data

The datasets analyzed in the current study were publicly available: https://dhsprogram.com/data/available-datasets.cfm

## Results

Previous surveillance studies are summarized in Table 1. Lunguya et al. collected the number of iNTS episodes in seven provinces in the Democratic Republic of Congo, and the proportion of iNTS (/1000) ranged from 0 (Kasai-Occidental) to 72 (Equateur) [20]. Marks et al. carried out surveillance studies in a multi-country setting [21], and three countries, where corresponding DHS datasets were available, were selected in the current study. The proportion of iNTS varied across the three sites with Asante Akim North in Ghana being the highest and Pikine in Senegal being the lowest. An additional surveillance study was conducted by Tapia et al. in Mali [22].

**Table 1:** Previous surveillance sites

Article	Country	Province	Site	Age group	iNTS episode^**e**^	Blood culture	Proportion of iNTS (per 1000 blood cultures)	Source
Lunguya et al.	Democratic Republic of Congo	Bas Congo	Kinsantu	Overall	114	2508	45.5	[20]
		Kinshasa	Kinshasa town	Overall	63	5499	11.5	
		Bandundu	Center^a^	Overall	1	73	13.7	
		Equateur	Bwamanda	Overall	29	403	72.0	
		Kasai-Occidental	Ilebo^a, b^	Overall	0	2	0.0	
		Kasai-Oriental	Center^a^	Overall	1	26	38.5	
		Orientale	Kisangani	Overall	18	1123	16.0	
Marks et al.^c^	Burkina Faso	Ouagadougou	Nioko2 & Polesgo^d^	Overall	60	1674	35.7	[21]
	Ghana	Asante	Asante Akim North	0–14.9 yo	145	2651	54.8	
	Senegal	Dakar	Pikine	Overall	4	1058	3.8	
Tapia et al.	Mali	Bamako	Bamako & Koulikoro	0–15.9 yo	667	26,126	25.5	[22]

^a^In Bandundu, Kasai-Occidental, and Kasai-Oriental, there were no fixed surveillance sites as samples were collected on purpose (i.e. suspicion of outbreaks). Thus, the centroids of the three provinces were used instead

^b^Given that there were only two blood cultures taken in Kasai-Occidental, it was possible to identify the location based on email correspondences with the authors of the article

^c^Among all countries reported by Marks et al. [21], the current table only shows the countries where corresponding DHS datasets are available. For other countries, see Marks et al. [21]

^d^The two sites in Burkina Faso were adjacent, thus a single set of geo-coordinates was used

^e^In the Democratic Republic of Congo, the number of iNTS cases was the sum of Typhimurium and Enteritidis reported by Lunguya et al. There were no adjusted iNTS cases available in Senegal, thus raw cases were applied

**Fig. 2:**
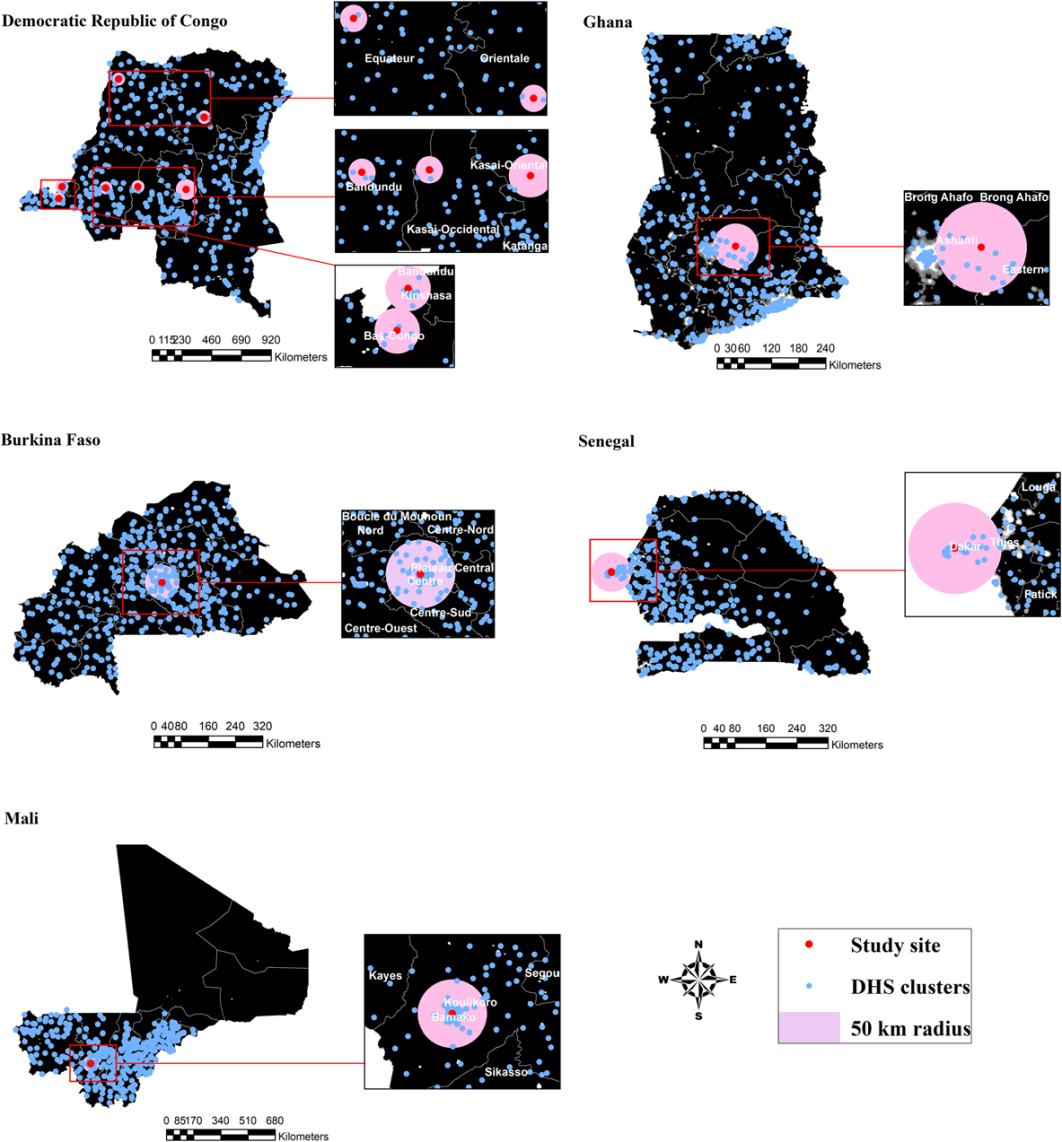
The selected DHS clusters per study site

Figure 2 shows how the number of clusters was selected to reflect improved locality around each of the existing study sites. Each cluster included 17 to 34 households where the number of household members in each household ranged from 1 to 49. The density of DHS clusters within a country varies depended on the size of a country and other logistical issues. For example, the dispersion of DHS clusters is relatively lower in Burkina Faso compared to the dispersion in the Democratic Republic of Congo as shown in Fig. 2. The iNRF indices with an equal weight, as well as with relative weights were first constructed based on the four risk factors distributed within the radius per study site.

**Fig. 3:**
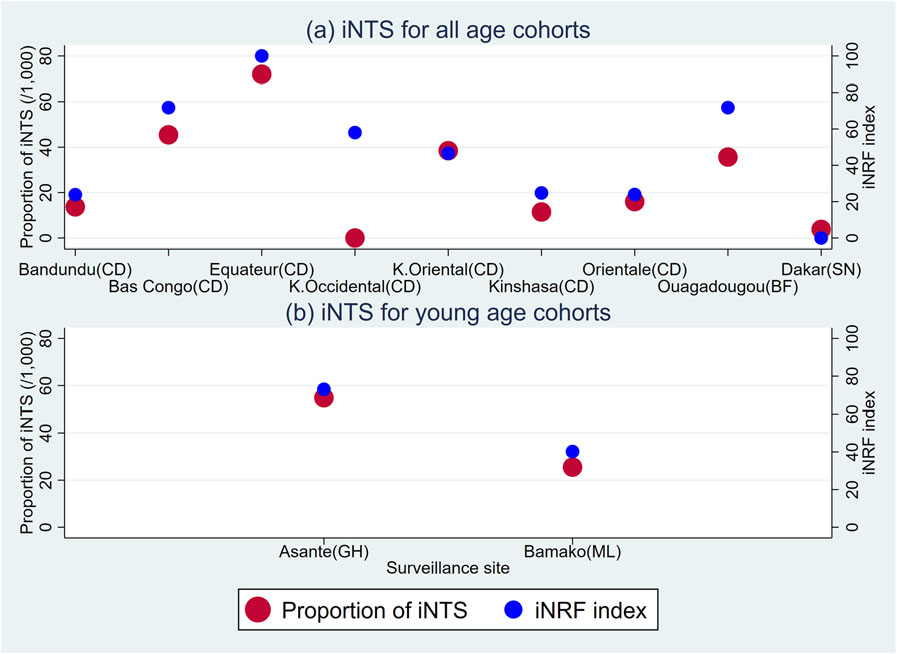
Correlation between the proportion of iNTS and the iNRF index. iNTS: Invasive Non-Typhoidal *Salmonella.* iNRF: iNTS Risk Factors. CD: The Democratic Republic of Congo, BF: Burkina Faso, SN: Senegal, GH: Ghana, ML: Mali

Correlations between the proportion of iNTS and the iNRF index with an equal weight were plotted in Fig. 3 (see Additional file 2 for the iNRF index with relative weights). A previous study indicated that young children were more vulnerable to *Salmonella* Typhimurium or *Salmonella* Enteritidis [20]. Given that the reported iNTS episodes were for younger age cohorts in Ghana and in Mali compared to other sites, these two studies were separately compared. Overall, the proportion of iNTS and the iNRF index with an equal weight was highly correlated showing the correlation coefficient of 0.821 at the 1% Bonferroni-adjusted significance level (*p* = 0.002). Nonetheless, the relationship between iNTS and the iNRF index in Kasai-Occidental appeared to be less consistent compared to that in other sites. The correlation coefficient with the iNRF index based on relative weights was 0.809 at the 1% Bonferroni-adjusted significance level (*p* = 0.003). Thus, the iNRF index with an equal weight was chosen for further analyses (simply named the iNRF index hereafter).

**Fig. 4:**
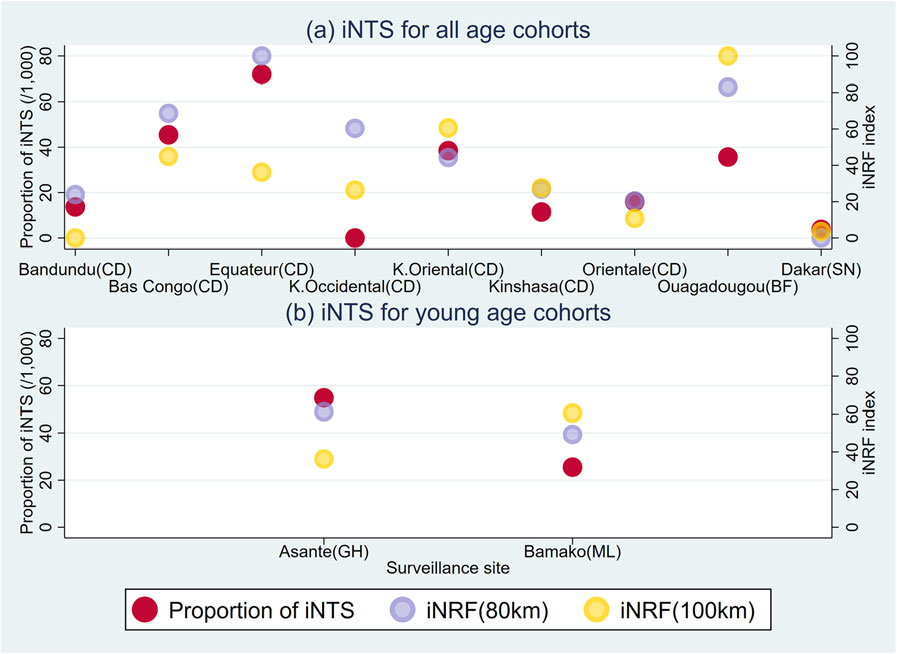
Sensitivity analyses with varying radii. iNTS: Invasive Non-Typhoidal *Salmonella.* iNRF: iNTS Risk Factors. CD: The Democratic Republic of Congo, BF: Burkina Faso, SN: Senegal, GH: Ghana, ML: Mali

Sensitivity analyses were conducted to examine how extending a radius would affect the association between the iNRF index and the proportion of iNTS. Figure 4 shows the relationship between the proportion of iNTS and the iNRF index estimated with additional radii: 80 km and 100 km. It is clear to see that the wider the radius is, the weaker the correlation is. The correlation coefficients were estimated at 0.755 (*p* = 0.007) and 0.629 (*p* = 0.038) with 80 km and 100 km radii, respectively. This result was consistent with the presumption that risk factors distributed in adjacent areas to a study site would improve the accuracy of the association with the proportion of iNTS.

**Fig. 5:**
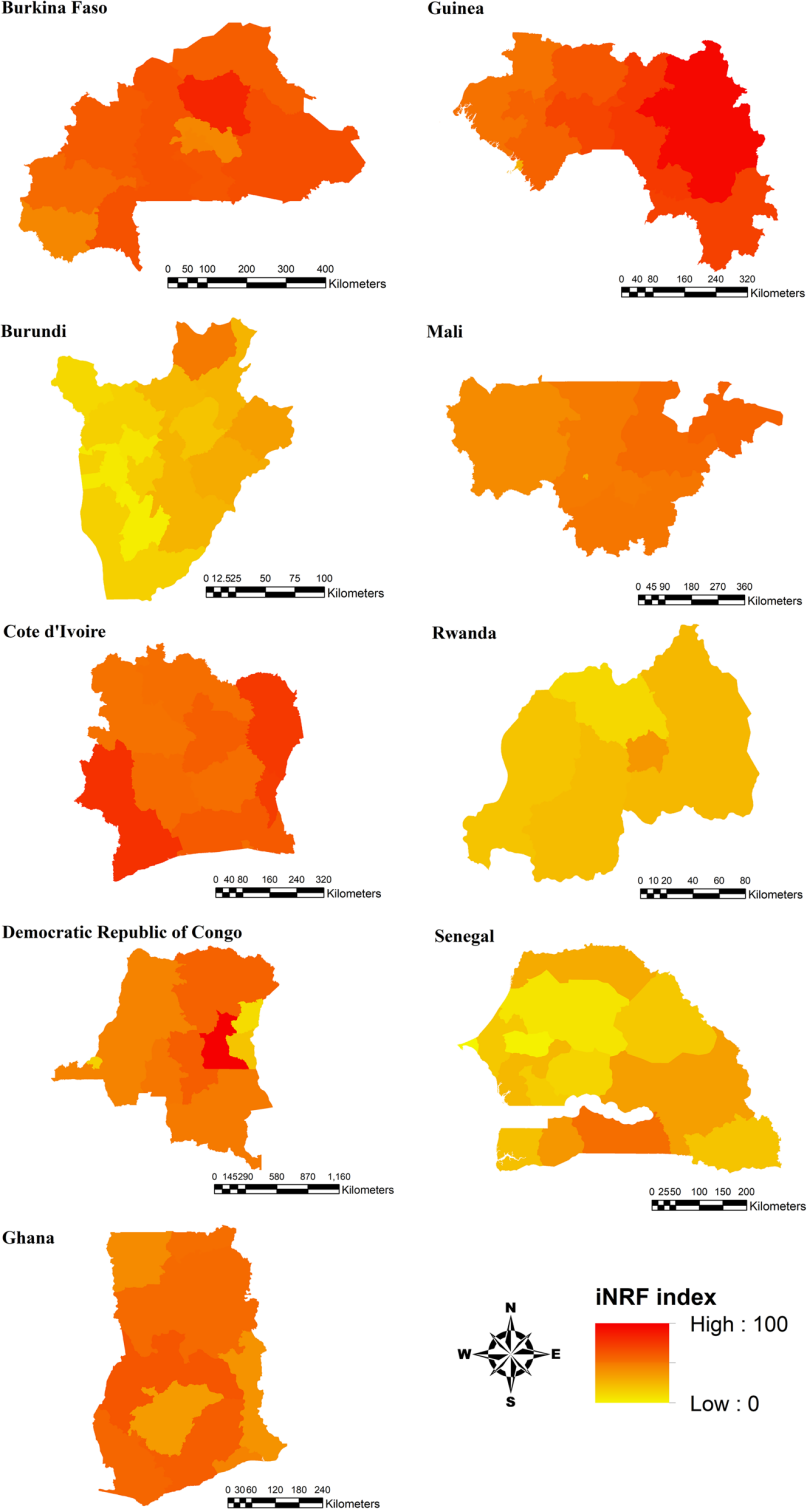
The geographical risk-level of iNTS in 9 countries

Given the association between the iNRF index and the proportion of iNTS episodes observed in the existing surveillance studies, the iNRF index was further estimated at the subnational boundary level for all 9 countries and shown in Fig. 5. At the country-level, Guinea appeared to be at the highest risk for iNTS followed by Cote d’Ivoire and Burkina Faso. On the other hand, Rwanda, Senegal, and Burundi were relatively at lower risk than other countries. At the provincial-level, the highest risk area was identified in Maniema, the Democratic Republic of Congo, whereas Dakar in Senegal was at the lowest risk. Supplementary table shows further details on individual index values and its percentiles at the subnational boundary level (see Additional file 1). The overall risk-level of iNTS was variable not only by country but also within a country. For example, while the iNRF index values were generally high (or low) across all provinces in Cote d’Ivoire (or Rwanda), the risk levels were variable by province in the Democratic Republic of Congo. The iNRF index values were low to moderate in some provinces such as Nord-Kivu, Sud-Kivu, and Kinshasa in the Democratic Republic of Congo, but the risk-level was much higher in Maniema and Ituri provinces in the same country.

## Discussion

The current study created a composite index called the iNRF index in order to identify potential high risk areas for iNTS. The iNRF index was generated based on four risk factors which were previously validated in literature, and the study outcome showed that the prevalence of iNTS was correlated to the iNRF index. In particular, the four risk factors used in this study were directly obtained from the survey data without imposing any unnecessary parametric assumptions for prediction. By using risk factors which were commonly available across countries, the risk-level of iNTS was estimated in a consistent manner at the subnational boundary level. The overall risk-level of iNTS was variable not only by country but also within a country, indicating that the actual burden of iNTS at the country-level would likely be different from a site-specific disease burden.

In the context of neglected tropical diseases where incidence rates are disproportionately high in developing countries, population-based surveillance data are scarce. In addition, data gathered in surveillance studies are mostly site-specific, e.g. within a single province of a country. Nonetheless, these site-specific surveillance data are often considered representative of an entire country and used for more complicated analyses such as global disease burden studies or cost-effective analyses. However, it is important to recognize that the burden of a disease is often variable not only between countries, but within a country. This variability within a country affects the overall disease burden estimate, as well as the cost-effectiveness of an intervention at the country-level.

iNTS has not yet been widely recognized compared to disease caused by *S.* Typhi or *S.* Paratyphi. Given the inadequacy of iNTS evidence, the current study constructed the iNRF index to understand the geographical distribution of iNTS risk factors in selected sub-Saharan African countries. While there is no doubt that population-based surveillance studies will provide more details on iNTS in the future, this kind of work is more resource and time intensive. Even if more surveillance studies became available, it would still be very difficult to obtain such information from all provinces within a country, especially in the developing-country setting. Thus, along with existing surveillance studies, the iNRF index presented in the current study can be used as a proxy to understand iNTS risk levels within a country and to support decision making for future iNTS research funding, clinical trial design/execution, and iNTS investment case development.

Some areas of uncertainty deserve attention. It should be noted that the 50 km radius used in this study is arbitrary. Nonetheless, the 50 km radius was chosen to ensure the minimum inclusion of DHS clusters in all countries given that the dispersion of DHS clusters was variable. In addition, the sensitivity analyses were conducted with varying radii and showed that the association between the iNRF index and site-specific iNTS estimates was more robust with a shorter radius than a longer radius. In general, while a longer radius would include more DHS clusters, it would weaken the precision to reflect the locality of study sites. On the other hand, a shorter radius would increase the precision of the estimate but reduce the number of cluster samples. Overall, the iNRF index was highly associated with the proportion of iNTS but did not correspond well with the observation in Kasai-Occidental. In Kasai-Occidental, there were only two blood cultures taken, making it difficult to generalize the outcome. Nonetheless, this may indicate that there might be additional risk factors which could explain the prevalence of iNTS. It should be noted that the number of adjusted iNTS episodes may have been estimated in a different way given logistical constraints. In fact, von Kalckreuth et al. indicated several challenges such as blood culture volume inadequacy or antimicrobial pretreatment which may have affected the limited diagnostic sensitivity of blood culture [23]. Identifying countries with a complete set of the four risk factors reduced the number of countries available for the current study. However, it was critical to include risk factors which had been frequently emphasized in existing literature (i.e., malaria and HIV) although these were not available in many of countries. On the other hand, focusing solely on risk factors which were more commonly available (i.e., drinking water source, or BMI) would increase the number of eligible countries, but these indicators alone were not particularly specific to iNTS but rather general, making the index less relevant to iNTS. This underscores the fact that it would be highly desirable for the DHS program to include all risk factors in more countries in the future. It is also worth noting that considering the current study aims at generating the index based on primary and standardized data across multiple countries, not all potential risk factors were included due to the absence of such data which met the inclusion standard. For example, sickle cell disease was also reported as a potential risk factor for iNTS [24] but not included due to the lack of available data. Nonetheless, having the four risk factors which met the strict inclusion criteria of the current study enabled us to generate robust outcomes to understand the endemicity of iNTS. Given the lack of existing surveillance data points and matching DHS datasets, it would be desirable to carry out more sophisticated statistical validation in the future as more population-based surveillance data and DHS countries with all four indicators will become available. Lastly, given the duration of a malaria episode is relatively short, the malaria indicator used in the current study might be less consistent with the actual level of burden.

Considering that a population-based surveillance study requires extensive human and financial resources, identifying potential high and low risk areas for iNTS prior to a study implementation would facilitate an appropriate site-selection process in the future. The iNRF index may also be used to adjust site-specific incidence rates when extrapolating them to other locations. It is worth noting that the accuracy of the global burden estimates can be further improved when employing risk factors directly obtainable at the subnational boundary level, rather than applying predicted values of risk factors with a set of assumptions.

## Conclusions

Population-based surveillance studies for iNTS are limited in number, and more surveillance studies are urgently needed. The insufficient number of data hampers the accurate estimation of the burden of iNTS and makes it difficult to understand the geographical variation of the disease. Because conducting surveillance studies in all specific locations within a country will be highly challenging in a short-run, the iNRF index presented in the current study can be used to make standardized comparisons of iNTS risk levels at the subnational boundary level where there is no surveillance data available yet. Moreover, the use of the iNRF index summarizing the four risk factors can help decision makers interpret easily and monitor the overall progress over time as the DHS program periodically updates survey data. Further research is needed not only to increase population-based surveillance data, but also to make the risk factors of iNTS available in more eligible countries.

## Supplementary Information

**Additional file 1.** Risk levels based on the iNRF index by Province.

**Additional file 2.** Correlation between the proportion of iNTS and the iNRF index with relative weights.

## Data Availability

The data obtained for our study is publicly available. The datasets generated and analyzed during the current study are available in the Demographic and Health Surveys, https://dhsprogram.com/.

## Abbreviations

iNTS: Invasive Non-Typhoidal *Salmonella*
iNRF: Invasive Non-Typhoidal *Salmonella* Risk Factors
DALYs: Disability-Adjusted Life Years
DHS: Demographic and Health Surveys
BMI: Body Mass Index
