# Supplementary material for: Geographical distribution of risk factors for invasive non-typhoidal Salmonella at the subnational boundary level in sub-Saharan Africa
Source: BMC Infect Dis. 2021 Jun 5;21:529. doi: 10.1186/s12879-021-06198-1 (PMC8180173; doi:10.1186/s12879-021-06198-1)
Supplement: Supplementary file 1 — Additional file 1. Risk levels based on the iNRF index by Province. [file 12879_2021_6198_MOESM1_ESM.docx]

**Risk levels based on the iNRF index by Province**

| **Country** | **Province** | **iNRF** | **Percentile** |
| --- | --- | --- | --- |
| Burkina Faso | Boucle de Mouhoun | 63.17 | 9 |
| Burkina Faso | Cascades | 44.89 | 5 |
| Burkina Faso | Centre | 45.81 | 6 |
| Burkina Faso | Centre-Est | 66.82 | 9 |
| Burkina Faso | Centre-Nord | 83.76 | 10 |
| Burkina Faso | Centre-Ouest | 66.06 | 9 |
| Burkina Faso | Centre-Sud | 64.25 | 9 |
| Burkina Faso | Est | 67.37 | 9 |
| Burkina Faso | Hauts Basins | 55.78 | 7 |
| Burkina Faso | Nord | 66.15 | 9 |
| Burkina Faso | Plateau Central | 43.93 | 5 |
| Burkina Faso | Sahel | 60.66 | 8 |
| Burkina Faso | Sud-Ouest | 64.98 | 9 |
| Burundi | Bubanza | 13.97 | 2 |
| Burundi | Bujumbura Mairie | 13.69 | 2 |
| Burundi | Bujumbura Rural | 3.91 | 1 |
| Burundi | Bururi | 2.43 | 1 |
| Burundi | Cankuzo | 34.32 | 4 |
| Burundi | Cibitoke | 11.49 | 1 |
| Burundi | Gitega | 26.35 | 4 |
| Burundi | Karusi | 19.99 | 3 |
| Burundi | Kayanza | 15.00 | 2 |
| Burundi | Kirundo | 48.62 | 6 |
| Burundi | Makamba | 13.75 | 2 |
| Burundi | Muramvya | 5.38 | 1 |
| Burundi | Muyinga | 26.86 | 4 |
| Burundi | Mwaro | 16.53 | 2 |
| Burundi | Ngozi | 24.01 | 3 |
| Burundi | Rumonge | 14.24 | 2 |
| Burundi | Rutana | 26.54 | 4 |
| Burundi | Ruyigi | 28.91 | 4 |
| Congo Dem Rep | Kwilu | 45.52 | 5 |
| Congo Dem Rep | Kongo Central | 45.42 | 5 |
| Congo Dem Rep | Tshuapa | 46.50 | 6 |
| Congo Dem Rep | Kasai-Central | 50.87 | 6 |
| Congo Dem Rep | Lomami | 60.52 | 8 |
| Congo Dem Rep | Tanganyka | 49.43 | 6 |
| Congo Dem Rep | Kinshasa | 13.85 | 2 |
| Congo Dem Rep | Maniema | 100.00 | 10 |
| Congo Dem Rep | Nord-Kivu | 9.69 | 1 |
| Congo Dem Rep | Ituri | 58.28 | 8 |
| Congo Dem Rep | Sud-Kivu | 18.49 | 3 |
| Cote d'Ivoire | Centre | 54.61 | 7 |
| Cote d'Ivoire | Centre-Est | 73.86 | 10 |
| Cote d'Ivoire | Centre-Nord | 61.11 | 8 |
| Cote d'Ivoire | Centre-Ouest | 55.51 | 7 |
| Cote d'Ivoire | Nord | 53.61 | 7 |
| Cote d'Ivoire | Nord-Est | 76.00 | 10 |
| Cote d'Ivoire | Nord-Ouest | 53.42 | 7 |
| Cote d'Ivoire | Ouest | 78.47 | 10 |
| Cote d'Ivoire | Sud sans Abidjan | 62.94 | 9 |
| Cote d'Ivoire | Sud-Ouest | 79.55 | 10 |
| Cote d'Ivoire | Ville D'Abidjan | 53.52 | 7 |
| Ghana | Ashanti | 36.61 | 5 |
| Ghana | Brong Ahafo | 63.78 | 9 |
| Ghana | Central | 60.17 | 8 |
| Ghana | Eastern | 60.97 | 8 |
| Ghana | Greater Accra | 46.66 | 6 |
| Ghana | Northern | 55.14 | 7 |
| Ghana | Upper East | 43.00 | 5 |
| Ghana | Upper West | 54.98 | 7 |
| Ghana | Volta | 40.15 | 5 |
| Ghana | Western | 56.79 | 8 |
| Guinea | Boke | 52.40 | 6 |
| Guinea | Conakry | 28.20 | 4 |
| Guinea | Faranah | 75.83 | 10 |
| Guinea | Kankan | 95.96 | 10 |
| Guinea | Kindia | 58.62 | 8 |
| Guinea | Labe | 64.26 | 9 |
| Guinea | Mamou | 71.25 | 10 |
| Guinea | N'Zerekore | 73.18 | 10 |
| Mali | Bamako | 25.48 | 4 |
| Mali | Kayes | 42.81 | 5 |
| Mali | Koulikoro | 49.53 | 6 |
| Mali | Mopti | 60.15 | 8 |
| Mali | Segou | 55.15 | 7 |
| Mali | Sikasso | 50.09 | 6 |
| Rwanda | East | 24.56 | 4 |
| Rwanda | Kigali City | 38.25 | 5 |
| Rwanda | North | 10.93 | 1 |
| Rwanda | South | 22.08 | 3 |
| Rwanda | West | 18.91 | 3 |
| Senegal | Dakar | 0.00 | 1 |
| Senegal | Diourbel | 1.32 | 1 |
| Senegal | Fatick | 23.94 | 3 |
| Senegal | Kaffrine | 13.41 | 2 |
| Senegal | Kaolack | 15.41 | 2 |
| Senegal | Kedougou | 18.99 | 3 |
| Senegal | Kolda | 54.56 | 7 |
| Senegal | Louga | 6.95 | 1 |
| Senegal | Matam | 17.14 | 2 |
| Senegal | Saint-Louis | 29.03 | 4 |
| Senegal | Sedhiou | 37.26 | 5 |
| Senegal | Tambacounda | 34.01 | 4 |
| Senegal | Thies | 17.28 | 3 |
| Senegal | Ziguinchor | 20.44 | 3 |
