# Supplementary figures and images for: Geographical distribution of risk factors for invasive non-typhoidal Salmonella at the subnational boundary level in sub-Saharan Africa
Source: BMC Infect Dis. 2021 Jun 5;21:529. doi: 10.1186/s12879-021-06198-1 (PMC8180173; doi:10.1186/s12879-021-06198-1)

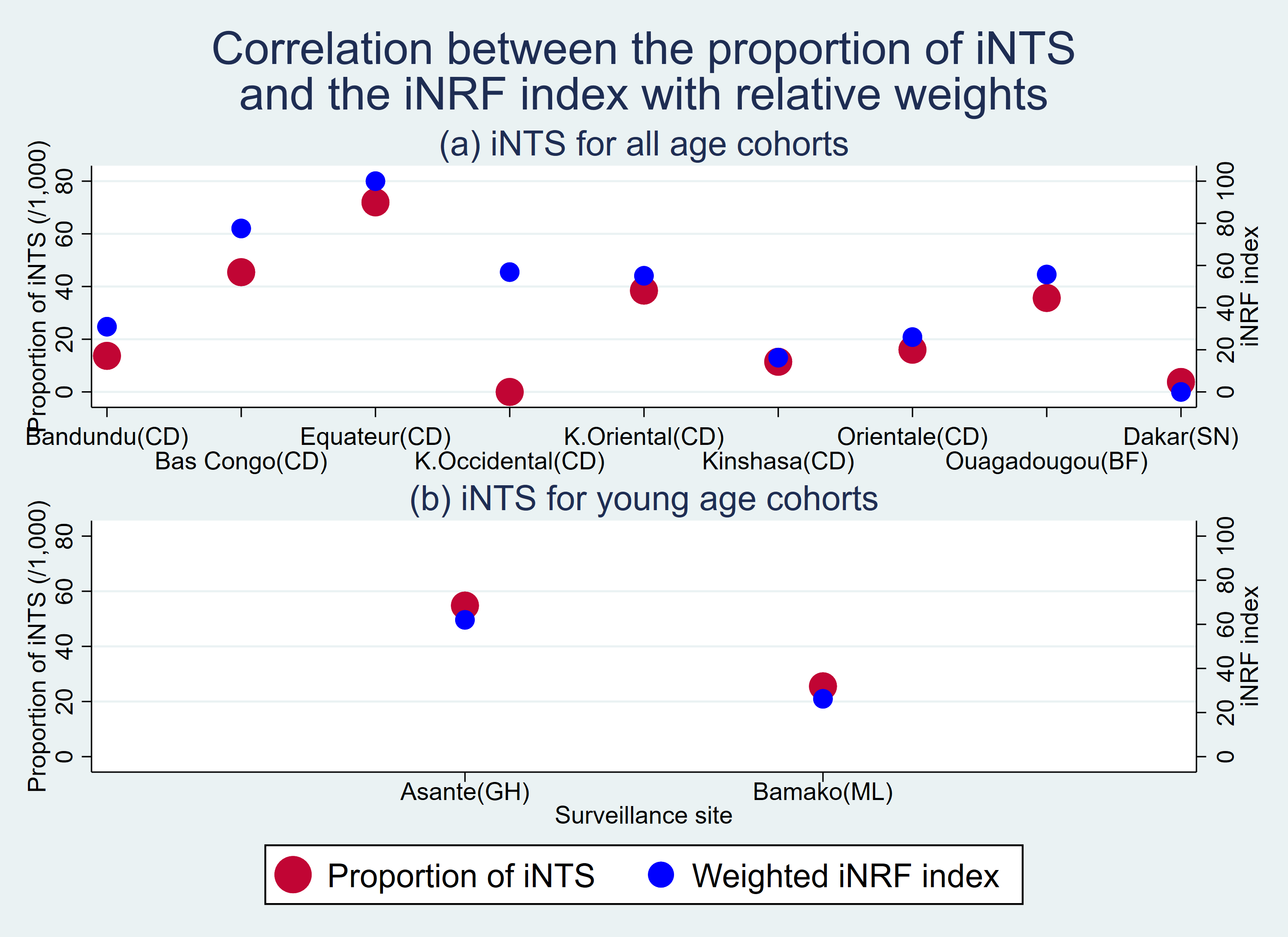

Supplement: Supplementary file 2 — Additional file 2. Correlation between the proportion of iNTS and the iNRF index with relative weights. [file 12879_2021_6198_MOESM2_ESM.tif]
